# The Synergic Role of Emerging and Endemic Swine Virus in the Porcine Respiratory Disease Complex: Pathological and Biomolecular Analysis

**DOI:** 10.3390/vetsci10100595

**Published:** 2023-09-27

**Authors:** Giovanni Pietro Burrai, Salwa Hawko, Silvia Dei Giudici, Marta Polinas, Pier Paolo Angioi, Lorena Mura, Alberto Alberti, Chadi Hosri, Georges Hassoun, Annalisa Oggiano, Elisabetta Antuofermo

**Affiliations:** 1 Department of Veterinary Medicine, University of Sassari, 07100 Sassari, Italy; gburrai@uniss.it (G.P.B.); salwa.hawko@hotmail.com (S.H.); alberti@uniss.it (A.A.); eantuofermo@uniss.it (E.A.); 2 Department of Animal Health, Istituto Zooprofilattico Sperimentale della Sardegna, 07100 Sassari, Italy; silvia.deigiudici@izs-sardegna.it (S.D.G.); pierpaolo.angioi@izs-sardegna.it (P.P.A.); lorena.mura@izs-sardegna.it (L.M.); annalisa.oggiano@izs-sardegna.it (A.O.); 3 Department of Veterinary Medicine, Faculty of Agricultural Sciences and Veterinary Medicine, Lebanese University, Beirut 1487, Lebanon; chadihosri@hotmail.com (C.H.); ghassoun@ul.edu.lb (G.H.)

**Keywords:** emerging viruses, pathology, RT-PCR, swine

## Abstract

**Simple Summary:**

Porcine respiratory disease complex (PRDC) represents a significant source of economic losses to pig farmers and swine industry worldwide, due to reduced growth, increased treatment costs and mortality rate. This disease complex is caused by various pathogens, including viruses such as PRRSV, PCV2, and bacteria such as *M. hyopneumoniae*. Co-infections of multiple agents, including emerging viruses such as TTSuV, PCV3, and PPV2, have been reported. These emerging viruses have been associated with lung lesions in pigs affected by PRDC. To investigate the presence of these agents, a study was conducted on swine lungs from different age groups. Histopathological examination and molecular tests were performed to identify involved agents. Results confirmed the presence of PPV2, TTSuV, and other viruses in affected pigs and revealed differences among swine age groups. The research provides valuable insights into PRDC’s complexity and highlights the importance of early detection and multidisciplinary approaches to effectively manage the disease in the intensive pig farming system.

**Abstract:**

Porcine respiratory disease complex (PRDC) represents a significant threat to the swine industry, causing economic losses in pigs worldwide. Recently, beyond the endemic viruses PRRSV and PCV2, emerging viruses such as TTSuV, PCV3, and PPV2, have been associated with PRDC, but their role remains unclear. This study investigates the presence of PCV2 and PRRSV and emerging viruses (PCV3, TTSuV, and PPV2) in the lungs of swine belonging to different age groups by histopathology and real-time PCR. The prevalent lung lesion was interstitial pneumonia with increased severity in post-weaning pigs. PRRSV was detected in 33% of piglets’ lungs and in 20% of adults and post-weaning pigs with high Ct, while PCV2 was found in 100% of adult pigs, 33% of post-weaning pigs, and 22% of piglets, with low Ct in post-weaning pigs. PCV3 was present in all categories and coexisted with other viruses. TTSuV was detected in all swine in combination with other viruses, possibly influencing the disease dynamics, while PPV2 was detected in 100% of adults’ and 90% of piglets’ lungs. The detection of TTSuV, PCV3, and PPV2 in affected pigs prioritizes the need for comprehensive approaches in implementing appropriate control measures and minimizing economic losses associated with PRDC.

## 1. Introduction

Porcine respiratory disease complex (PRDC) is a multifactorial syndrome that represents an important threat to pig farmers and producers, being one of the main sources of global swine industry loss [1,2,3]. It causes a reduction in growth and an increase in treatment costs and mortality rates, negatively impacting the pork industry and intensive pig farms worldwide [4,5]. The pig’s respiratory tract is vulnerable to various pathogenic agents (viruses, bacteria, parasites) causing lesions due to either primary infection (capable of evading immune systems) or secondary infections (host infection after primary invasion) [6]. Swine pneumonia prevalence varies from 20% to 80% among different areas, production systems, and lesion-scoring systems used [1,2,3,4,5,6]. The most relevant agents in PRDC are porcine respiratory and reproductive syndrome virus (PRRSV), porcine circovirus 2 (PCV2), and bacteria (*M. hyopneumoniae*) [1,2,4].

Generally, co-infection with different swine pathogens is common in intensive systems, and reports of coexisting agents in the respiratory system are recently increasing, despite the limited knowledge about their specific role [1,2,7,8]. PRRS, PCV2, and other emerging viruses’ co-infection has been an important factor in PRDC [7,9]. Swine emerging viruses causing significant lung lesions and impacting on the swine industry worldwide are torque teno sus virus (TTSuV), porcine circovirus 3 (PCV3), and parvovirus (PPV) 2 [10,11]. Although most emerging viruses are ubiquitous in global pig populations, knowledge of their biology, pathogenicity, and possible health threats is still lacking [12,13,14].

TTSuV (*Anelloviridae, Anellovirus*) is a non-enveloped virus first reported in 1999, with a circular, small, single-stranded, negative-sense DNA genome [15,16,17]. TTSuV transmits horizontally via a fecal–oral route, vertically and sexually, as demonstrated by the presence of TTSuV in boars’ semen [18,19,20]. Mixed transmission routes promote worldwide spreading of TTSuV [19,21,22]. Although TTSuV causes lung lesions in pigs affected by PRDC, macroscopic and microscopic lesions caused by this virus are still underinvestigated [23,24,25,26,27].

PCV3, first identified in 2015 in a sow farm with porcine dermatitis and nephropathy syndrome (PDNS) and reproductive failure, is a non-enveloped virus with a circular, small, single-stranded DNA genome, belonging to the family *Circoviridae*, genus *Circovirus* also including PCV1, PCV2, and PCV4 [28,29,30,31,32]. PCV3 is transmitted both horizontally and vertically and can undergo cross-species transmission [31,33]. It has been found in domestic and wild pigs, with the highest prevalence in wild boars playing a very important role in its epidemiology as reservoirs [34]. A study on pigs naturally infected with PCV2 and PCV3 reported interstitial pneumonia as a common lesion [35]. 

PPVs are members of the *Parvoviridae* and are linear, small, non-enveloped, negative-sense, single-stranded DNA viruses [36,37]. PPV2 and PPV3 were first discovered in 2001 and 2007, respectively, whereas PPV4 was detected in 2010 ([38] and references therein). PPV5 was detected in 2013, PPV6 in 2014, and PPV7 in 2016 [37,38,39,40]. Recent investigations reported a significant association between interstitial pneumonia and PPV2, without other swine pathogens (viruses and bacteria) of clinical significance [33,38]. 

In 2018, the presence of the new viruses PPV2, PPV3, PPV6, and TTSuV1a was reported in China in naturally infected piglets presenting respiratory syndrome. The strong association of these emerging viruses to PRDC complicated the pathogenesis of this syndrome [9]. Consequently, a conclusive PRDC diagnosis is challenging and needs both evaluation of gross and microscopic lesions, and the detection of causative agents implicated in disease progression [5,9].

Consequently, this work aims to investigate commonly reported (PCV2, PRRSV) and emerging viruses (PCV3, TTSuV, PPV2) by real-time PCR and the macroscopical and histopathological features of swine lungs affected by PCDC.

## 2. Materials and Methods

### 2.1. Sample Collection

Swine lungs (29) belonging to three different age groups of hybrid-breed pigs ([10] adult pigs (174 days), 10 post-weaning (65 days), and 9 piglets (40 days)) were collected from a North Sardinian slaughterhouse and macroscopically evaluated at the Department of Veterinary Medicine of Sassari University.

All swine came from one close-breeding farm and showed mild clinical signs mostly consisting of coughing and dyspnea, reduced feed intake, and weight loss. Piglets were immunized against *Mycoplasma hyopneumoniae* and PCV2, post-weaning for *Mycoplasma hyopneumoniae*, Pseudorabies disease, and PCV2, whereas adult pigs were vaccinated against *Mycoplasma hyopneumoniae*, pseudorabies disease, PCV2, *Erysipelothrix rhusiopathiae*, *Parvovirus, E. coli* and atrophic rhinitis disease.

Two specular aliquots were collected, the first for histological evaluation, while the other was submitted to the Istituto Zooprofilattico Sperimentale della Sardegna (IZS) for downstream molecular analysis.

### 2.2. Histopathology

Lungs were 10% neutral buffered formalin-fixed and then paraffin-embedded. Tissue sections of 3 μm in thickness were hematoxylin and eosin (HE) stained. Samples were evaluated by histopathology, based on the morphological tissue changes, type, distribution, and severity of the inflammatory lesions.

### 2.3. DNA/RNA Extraction and Real-Time PCR (RT-PCR)

The second lung aliquot submitted to the IZS was prepared for DNA/RNA extraction performed with MagMAX^TM^ CORE nucleic acid purification kit and the MagMax96 extractor (Thermo Fisher, Waltham, MA, USA) according to manufacturer instructions [41]. RT-PCR assays were performed to detect the genome of PRRSV, PCV2, PCV3, TTSuV, and PPV2 using different conditions. PRRSV RT-PCR was performed using the commercial kit LSI VetMax PRRSV EU/NA 2.0 lit (Life Technologies, Carlsbad, CA, USA) according to the manufacturer’s instructions. PCV2 RT-PCR was performed with cycling parameters of 10 min at 95 °C; 40 cycles of 15 s at 95 °C, and 60 °C for 1 min. The PCR reaction consisted of 12.5 µL of the Taqman Fast Advanced Master Mix (Applied Biosystems, Woburn, MA, USA), 0.2 µM of the probe, 0.9 µM of each forward and reverse primers, and 5 µL of sample DNA in a final volume of 25 µL [42]. PCV3 RT-PCR was performed using the following conditions: 95 °C for 10 min, 45 cycles of 95 °C for 10 s, and 60 °C for 30 s. The protocol of the PCR assay was as follows: 5 µL of the Taqman Fast Advanced Master Mix, 2 µL of the extracted DNA, 0.6 µM of the probe, 0.3 µM of primers, and sterile water added to reach a final volume of 10 µL [43]. TTSuV RT-PCR assay was performed following the conditions: 95 °C for 10 min; 50 cycles of 95 °C for 15 s, and 60 °C for 60 s. The PCR assay master mix was composed of 2 µL of extracted DNA and 5 µL of Taqman Fast Advanced Master Mix, 0.6 µM of each forward and reverse primers, 0.3 µM of the probe in a final volume of 10 µL [44]. PPV2 RT-PCR was performed under the conditions, as follows: 10 min at 95 °C; 45 cycles of 10 s at 95 °C, and 60 °C for 30 s. The assay was performed in a final volume of 10 µL, composed of 2 µL of extracted DNA, 5 µL of the Taqman Fast Advanced Master Mix, 0.6 µM of each primer, and 0.3 µM of the probe [45].

Primers and probes used are reported in Table 1.

### 2.4. Sequencing

Sanger sequencing was performed to confirm the specificity of the real-time for the emerging viruses PPV2 and TTSuV and, given the high threshold cycle (Ct) values, to verify the PRRSV circulation in the farm. Two samples from an adult and a post-weaning pig and three samples from two adults and a piglet showing a Ct value < 25 were analyzed for PPV2 and TTSuV, respectively. Instead, a postweaning pig with a Ct value of 31, was examined for PRRSV.

Sequencing was performed in both senses at the IZS using the primers reported in Table 2 and a DNA sequencing kit (dRhodamine Terminator Cycle Sequencing Ready Reaction; Applied Biosystems) on an ABI-PRISM 3500 Genetic Analyzer (Applied Biosystems, Waltham, MA, USA). BioEdit software version 7.0.0 was used to edit sequences and Blastn (https://blast.ncbi.nlm.nih.gov, accessed on 18 July 2023) for their identification. 

### 2.5. Statistical Analysis

Age and categories of swine (adults, post-weaning, and piglets), histopathological, and RT-PCR results were registered and submitted for statistical analysis.

Microscopical results and RT-PCR data were analyzed using Stata 11.2 and Minitab 15 software (StataCorp LP, College Station, TX, USA; Minitab, Ltd., Coventry, United Kingdom). In particular, histological and RT-PCR data were examined with the non-parametric Kruskal–Wallis test following Dunn’s post hoc comparison. A *p*-value < 0.05 was considered significant.

## 3. Results

### 3.1. Macroscopical Findings

**Figure 1:**
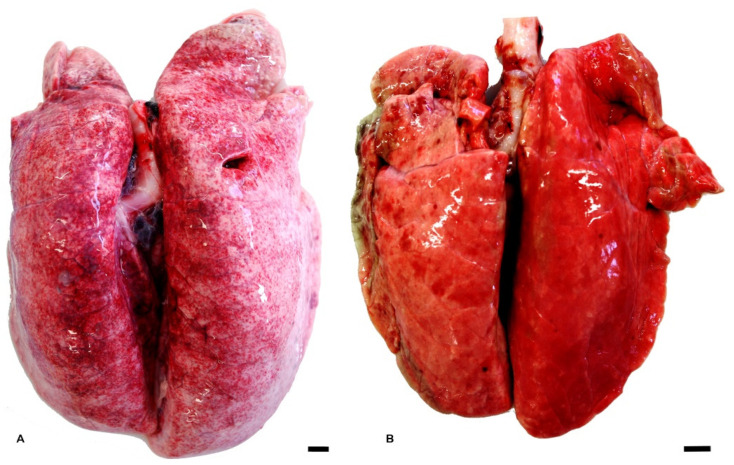
Lung macroscopical findings. (**A**): moderate diffuse interstitial pneumonia with rib impression in a lung of a post-weaning pig. (**B**): cranioventral moderate chronic fibrinous and suppurative bronchopneumonia in a piglet. Bar: 1 cm.

Lungs showed multifocal hyperemic areas associated with an increase in the volume of the parenchyma in 10/10 (100%) adults. Evidence of ribs impression was found in 9/9 (100%) piglets, and in 10/10 (100%) post-weaning suggestive of diffuse interstitial pneumonia (Figure 1A). A moderate, locally extensive chronic fibrinous and suppurative bronchopneumonia of the cranial ventral margins was detected in 1/9 of piglets (11%) (Figure 1B). Moreover, abundant whitish-foamy fluid was observed in the lumen of the trachea and the main bronchi. 

### 3.2. Histological Findings

#### 3.2.1. Adult Pigs

**Figure 2:**
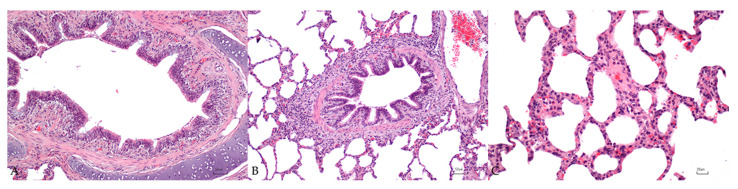
Lung microscopical findings in adult swine. (**A**): mild chronic lymphoplasmacytic and macrophagic bronchitis. (**B**): moderate hyperplasia of the bronchus-associated lymphoid tissues. (**C**): mild chronic interstitial pneumonia. Bar: 50 µm (**A**,**B**) and 20 µm (**C**).

In the lamina propria of the bronchi, 7/10 (70%) adult pigs presented chronic mild and diffuse lymphoplasmacytic and eosinophilic inflammatory infiltrate. In particular, in 4/7 (57%) pigs, the infiltrate was characterized by a scarce number of lymphocytes, plasma cells, and macrophages and was classified as chronic lymphoplasmacytic and macrophagic bronchitis, and in 3/7 (43%) pigs the infiltrate was mainly composed of eosinophils (Figure 2A). Moderate and diffuse hyperplasia of the bronchus-associated lymphoid tissues (BALT) was detected in 2/10 (20%) pigs, showing lymphoplasmacytic and macrophagic bronchitis (Figure 2B). A multifocal to diffuse chronic interstitial pneumonia was observed in 6/10 (60%) pigs characterized by a mild (3/10; 30%) to moderate (3/10; 30%) inflammatory infiltrate of lymphocytes, macrophages, and plasma cells expanding the interstitium (Figure 2C). Furthermore, in 2/6 (33%) pigs the alveoli showed mild destruction of alveolar septa with enlargement of alveolar spaces (emphysema).

#### 3.2.2. Post-Weaning Pigs

**Figure 3:**
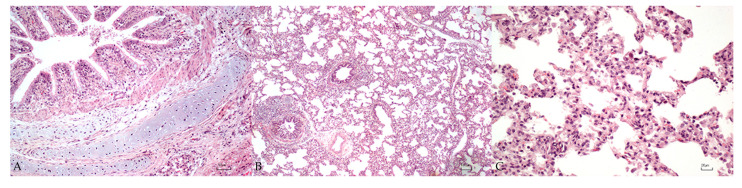
Lung microscopical findings in post-weaning swine. (**A**): mild chronic lymphoplasmacytic and macrophagic bronchitis. (**B**): mild chronic lymphoplasmacytic and macrophagic bronchiolitis with moderate hyperplasia of the bronchus-associated lymphoid tissues. (**C**): moderate chronic interstitial pneumonia. Bar: 50 µm (**A**), 100 µm (**B**) and 20 µm (**C**).

A mild chronic lymphoplasmacytic and macrophagic bronchitis was detected in 4/10 (40%) post-weaning pigs, characterized by a multifocal inflammatory infiltrate of lymphocytes, plasma cells, and macrophages in the lamina propria of the bronchi (Figure 3A). Bronchioles in post-weaning pigs were only mildly affected, presenting mild multifocal necrosis of the epithelium in 3/10 (30%) pigs (Figure 3B). Histopathological changes in the alveolar septa, consisting of loss of type Ⅰ alveolar cells and hypertrophy of type Ⅱ alveolar cells, were detected in 3/10 (30%) pigs. A low number of macrophages was detected in the lumen of the alveoli in 4/10 (40%) pigs, whereas a moderate number of erythrocytes was found in the alveolar space of 1/10 (10%) pigs. Mild to moderate multifocal emphysema was observed in 3/10 (30%) pigs. Noteworthy, the main histopathological finding was the presence of a chronic multifocal to diffuse interstitial pneumonia in 10/10 (100%) post-weaning pigs, mostly characterized by moderate (7/10; 70%) to severe (3/10; 30%) inflammatory infiltrate of lymphocytes, macrophages, and plasma cells expanding the interstitium (Figure 3C).

#### 3.2.3. Piglets

**Figure 4:**
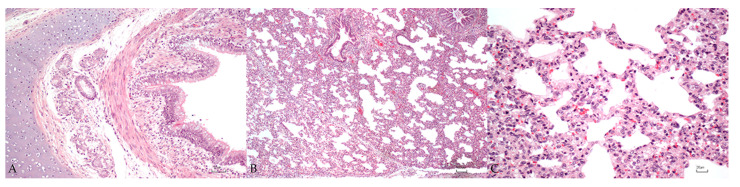
Lung microscopical findings in piglets. (**A**): mild chronic lymphoplasmacytic and macrophagic bronchitis. (**B**): mild chronic lymphoplasmacytic and macrophagic bronchiolitis associated with diffuse severe interstitial pneumonia. (**C**): severe chronic interstitial pneumonia. Bar: 50 µm (**A**), 100 µm (**B**) and 20 µm (**C**).

A mild chronic lymphoplasmacytic and macrophagic bronchitis was detected in 8/9 (89%) piglets, characterized by a multifocal to diffuse infiltrate of lymphocytes, plasma cells, and macrophages in the lamina propria of bronchi (Figure 4A). In the bronchi, a mild to severe detachment of epithelial cells was also detected in 3/9 piglets (33%). At the level of the lamina propria of bronchioles a multifocal to diffuse chronic lymphoplasmacytic and macrophagic bronchiolitis was detected in 8/9 piglets (89%), characterized by a mild (7/8) to moderate (1/8) infiltrate of lymphocytes, plasma cells, and macrophages (Figure 4B). The main histopathological finding in piglets was chronic diffuse interstitial lymphoplasmacytic and macrophagic pneumonia in 9/9 (100%) piglets, characterized by moderate (2/9) to severe (7/9) inflammatory infiltrates of lymphocytes, plasma cells, and macrophages that expanded the interstitium, often associated with the presence of extravasated erythrocytes (8/9) (Figure 4C).

### 3.3. Real-Time PCR Results

#### 3.3.1. Adult Pigs

PCV2 was detected in 10/10 adult pigs (100%) with a mean (±SD) threshold cycle (Ct) value of 27.22 ± 4.43, while the lungs of 2/10 pigs (20%) hold the PRRS viral genome. In particular, the PRRS-EU strain expressed a mean Ct value of 36.59 ± 0.41, whereas no positivity was found for the PRRS-NA strain.

PCV3 was detected in 1/10 adult pigs (10%) with a mean Ct value of 19.91, while all pigs’ lungs (10/10) (100%) were found positive for PPV2 with a mean Ct value of 27.29 ± 1.12. Lastly, TTSuV was detected in 10/10 adult pigs (100%) with a mean Ct value of 25.38 ± 3.10 (Table 3).

#### 3.3.2. Post-Weaning Pigs

PCV2 was detected in 3/10 (33%) post-weaning pigs with a mean Ct value of 24.05 ± 9.7, while the lungs of 2/10 (20%) hold the PRRS-EU strain with a mean Ct value of 34.30 ± 4.35, whereas no positivity was found for the PRRS-NA strain.

PCV3 was detected in 2/10 (20%) pigs with a mean Ct value of 29.07 ± 12.15, while PPV2 was detected in 5 pigs out of 10 (50%) with a mean Ct value of 21.21 ± 5.47. All lungs (100%) resulted positive for TTSuV with a mean Ct value of 29.39 ± 2.98 (Table 3).

#### 3.3.3. Piglets

PCV2 was detected in 2/9 (22%) piglets with a mean Ct value of 29.67 ± 8.84, while the lungs of 3/9 (33%) piglets presented PRRS-EU strain with a mean Ct value of 37.38 ± 2.13, whereas, for the strain PRRS-Na, 11% (1 piglet out of 9) showed positivity with a Ct value of 36.94.

PCV3 was detected in 4/9 (44%) piglets with a mean Ct value of 35.59 ± 4.88. PPV2 was detected in 8/9 (89%) piglets with a mean Ct value of 23.06 ± 4.83, while 9/9 lungs (100%) resulted positive for TTSuV with a mean Ct value of 30.13 ± 5.32 (Table 3).

### 3.4. Sequencing 

Sample sequencing confirmed the positivity for PPV2, TTSuV, and PRRSV. The Blast analysis showed the highest similarity (97%) of PPV2 sequences with two Chinese strains collected in 2009 (GenBank accession number GU938299) and 2017 (MK378208). TTSuV sequences obtained in this study revealed the circulation of both TTSuV1 and TTSuVk2, in the analyzed farm. TTSuV1 sequences had the highest similarity (98%) with strains from Brazil (KX833781) and South Korea (JF451485). Instead, TTSuV2k sequences had 98% identity with strains from China collected in 2015 (MK263732) and 2016 (MK263716). A sample from an adult pig was confirmed to be coinfected with PPV2, TTSuV1, and TTSuVk2. The PRRSV sequence had the highest similarity (96%) with an Italian strain (OL699297) collected in 2018.

### 3.5. Statistical Analysis

Histological results were compared using the non-parametric Kruskal–Wallis test with Dunn’s post hoc comparison, considering the intensity x distribution of the inflammation. The histological evaluations highlight a statistically significant difference between the severity of the lesions in the different swine categories. In particular, bronchiolar lesions were more severe in adults and piglets than in post-weaning pigs (Kruskal–Wallis test, χ^2^ chi-square 13.771; *p* < 0.05), while there was no statistically significant difference between adult pigs and piglets (*p* > 0.05). Post-weaning pigs showed more severe lesions in the lung interstitium than adults (Kruskal–Wallis test, χ^2^ 12.304; *p* < 0.05), while no statistically significant differences were noticed between adult pigs and piglets (*p* > 0.05). PCR results were compared among the three different animal categories using the non-parametric Kruskal–Wallis test with Dunn’s post hoc comparison. A statistically significant difference was noticed between the Ct values of PPV2 and TTSuV in the different swine groups. In particular, post-weaning and piglets have a lower PPV2 mean Ct value compared to adults (Kruskal–Wallis test, χ2 chi-square 7.116; *p* < 0.05), while adults have TTSuV Cts compared to post-weaning and piglets (Kruskal–Wallis test, χ^2^ chi-square 7.836; *p* < 0.05). No other significant differences were observed (*p* > 0.05).

## 4. Discussion

PRDC is a multifactorial respiratory disease that causes significant economic losses in the swine industry due to the reduction of growth rates, the rise of medication costs, and the increase in mortality rates [48]. PRDC is caused by combined viral, bacterial, and environmental factors, which act synergistically to compromise the pig’s respiratory system [1,4]. 

Recently, a significant number of viruses have been claimed to be involved in the development of this disease [1,2,3]. PRRSV and PCV2 have been historically described as lesions’ triggers while emerging ones such as PCV3, PPV2, and TTSuV have rarely been reported [49]. Thus, in this work, we explore macroscopical and histopathological lung lesions in different swine age categories and we investigate the presence of the historically reported viruses (PRRSV and PCV2) and emerging ones (PCV3, PPV2, TTSuV).

Macroscopical findings revealed lung rib impressions, indicative of diffuse interstitial pneumonia, and suggested a widespread respiratory disease mostly affecting the younger classes of pigs. In 1/9 piglets, moderate chronic fibrinous and suppurative bronchopneumonia was observed, indicating the presence of localized respiratory infection.

Histopathology showed chronic mild bronchitis and bronchiolitis (70% to 90% respectively) associated with chronic interstitial pneumonia in 60% of adult pigs. Post-weaning pigs exhibited chronic interstitial pneumonia (100%), whereas mild chronic bronchitis was present in 40% of the animals, while in piglets chronic moderate to severe interstitial pneumonia was detected in 100% of animals, associated with mild chronic bronchitis and bronchiolitis in 89% of the piglets. Adult pigs and piglets were characterized by more severe bronchiolar lesions than post-weaning pigs (*p* < 0.05), whereas the latter showed more severe lesions in the lung interstitium than adults (*p* < 0.05). As reported by Ruggeri and coauthors, lung lesions are one of the most frequent lesions affecting piggeries worldwide [2]. Moreover, our data slightly differ from what has been reported regarding the association between the different types of lesions (i.e.,: catarrhal bronchopneumonia, purulent bronchopneumonia, interstitial pneumonia, etc.) and the swine categories, being interstitial pneumonia more frequently recorded by other authors in growing/fattening pigs than post-weaning. This difference could be related to the different adopted protocols based on macroscopical evaluation alone or, as in our case, by joint macroscopical and histological description [2].

Samples were also tested by RT-PCR for emerging swine viruses such as PCV3, PPV2, TTSuV, and two of the most common viruses in pig farming (PCV2 and PRRSV).

PRRSV was detected in 33% of lungs in piglets and 20% in each of the adult and post-weaning pig categories. Furthermore, the high Ct values detected, ranging from 34.30 to 37.38, were indicative of a low viral load and suggested a low presence of PRRSV in the examined samples.

PCV2 was detected in 100% of adult pigs’ lungs, in 33% of post-weaning, and in 22% of piglets, with a low threshold cycle post-weaning. As reported, immunization against PCV2 can reduce the mortality rate by limiting viremia, virus spreading, and the onset of pathological lesions ([50] and references therein). Conversely, there have been reports indicating PCV2 vaccination failures, leading to the manifestation of clinical symptoms associated with PCVD [51,52,53,54]. A study on PCV2 reported that the lung lesions caused by PCV2 consist of granulomatous interstitial pneumonia associated or not with bronchiolitis and bronchiolar fibrosis [55]. Another recent review reported that PCV2 infection causes interstitial granulomatous pneumonia characterized by the expansion of peribronchial space and alveolar septa by macrophages and lymphocytes [1]. In addition, interstitial pneumonia in PCV2 and PRRSV infections often share similarities with overlapping morphological features [1]. In this study, it seems plausible that the observed lung lesions characterized by interstitial pneumonia are more related to PCV2 infections, detected cumulatively in 51% of pigs, than to PRRSV (24%). Furthermore, in comparison to PRRSV, the lower PCV2 Cts in all the examined categories suggested a significant involvement of this pathogen. The simultaneous presence of different viruses causing pulmonary involvements is frequent in swine herds and complicates diagnosis. As suggested by Sarli and co-workers, localization of the causative agents by additional methods such as immunohistochemistry (IHC) or in situ hybridization (ISH) within the lesions is a key point to understanding the etiopathogenesis of the disease and confirming the individual contribution of those pathogens in lung lesions [1].

PCV3 was detected in the lungs of all swine categories, mainly in piglets (44%) and to a lesser extent in post-weaning (20%) and adult pigs (10%). Interestingly, as reported by Klaumann and coauthors, PCV3 DNA has been detected in all age categories with a similar frequency and, similarly to our results, the highest PCV3 positivity has been detected in animals after weaning ([56,57] and references therein). Intriguingly, similar to our study, previous reports showed a frequent coexistence of PCV3 with other viruses (i.e., PCV2, PRRSV, PEDV, PPV, TTSuV1 and 2, PDCoV) ([41,57] and references therein). It was postulated that PCV3 alters the pig immune responses similarly to PCV2, causing immunosuppression [41]. Furthermore, it is still unclear if PCV3 could have a role as a secondary agent in immunocompromised pigs, or whether its infective potential is unrelated to the host immune system status [57] and references therein [58].

Regarding PCV3 pathogenicity, it has been demonstrated that this virus could infect swine, but no associated macroscopical lesions were described [49]. More recently, De Conti and coauthors described the association of histological lesions in different tissues with viral transcriptional activity [35]. More in detail, as in our samples, the authors identified, in cases with interstitial pneumonia, PCV3 mRNA in the alveolar septa suggesting that PCV3 could be an important swine pathogen with a potential role in swine lung disease.

Accordingly, further studies involving a greater number of samples, based on ISH to detect viral DNA and IHC with PCV3-specific monoclonal antibodies, are mandatory to demonstrate the real incidence of PCV3 in swine herds and to clarify its pathogenic role during co-infection.

In our study, TTSuV was detected in 100% of all age categories of swine. These data mirror several worldwide reports and could be ascribed to the ability of *Anellovirus* to use alternative transmission routes (fecal–oral, vertical, and transplacental/intra-uterine). Additionally, TTSuV was found in boars’ semen, suggesting a contribution to transmission via the sexual route ([49] and references therein). Furthermore, adult pigs showed lower TTSuV threshold cycles compared to post-weaning, and piglets, suggesting, as previously reported, that the viral load increases with the age of the animals [59,60,61]. Altogether, our results substantially support a progressive infection from a young age that persists throughout life and an inefficient immune response against the virus in the oldest animals.

Interestingly, a high prevalence of TTSuV1 and TTSuVk2 was reported in the lung and spleen in co-infection with PCVs [61,62]. Similarly, the contemporary presence of both TTSuV1 and TTSuVk2 was observed in our cases by Sanger sequencing. TTSuV1 sequences had the highest similarity with strains from Brazil and South Korea, while TTSuVk2 sequences had 98% identity with strains from China, suggesting a global and widespread diffusion of those viruses [61]. Notably, it is not clear whether TTSuV infection could be a primary agent of disease, as it is detected in both healthy and affected pigs at comparable frequencies [11,49]. Studies that investigate the macroscopic and microscopic aspects of TTSuV infection are scarce, and the correlation between the virus and lesions is still not fully demonstrated ([49] and references therein). Progressive and severe interstitial pneumonia was detected, among other lesions, in pigs experimentally infected with TTSuV [23,24,25,26]. In our study, a multifocal to diffuse chronic macrophagic and lymphoplasmacytic interstitial pneumonia was observed in 60% of adult pigs, in 100% of post-weaning pigs, and in 100% of piglets, suggesting a possible involvement of TTSuV in lung lesions. Furthermore, TTSuV has been found in combination with PCV2, PRRSV, and PPV, leading to difficulties in distinguishing the contribution of each virus to pathological features [18,63,64,65]. TTSuV may have a role in impairing the immune system and affecting disease dynamics [18]. In addition, and despite the limited data on gross and histopathological lesions caused by TTSuV and PCV3, these two viruses are related to several lesions, specifically to interstitial pneumonia [26,28,29,31]. Overall, based on our data, it seems plausible that TTSuV may play a role in lung swine disease, but further studies are needed to clarify if *Anellovirus* may enhance the severity of the disease caused by other swine pathogens.

PPV2 was detected in 100% and 90% of adults and piglets’ lungs, and to a lesser extent in post-weaning pigs. Variable prevalence of PPV2 has been reported by several epidemiological studies. PPV2 was found within alveolar macrophages, in 28% of lung samples presenting interstitial pneumonia, suggesting that the virus is associated with, but may not be the only agent responsible for, the PRDC ([27] and references therein). Overall, PPV2 has been recently reported in the literature, but its viral pathogenicity is still to be clarified, being detected in both affected and unaffected animals. A retrospective histopathological study demonstrated that PPV2 was associated with interstitial and broncho-interstitial pneumonia [38], while other authors considered unclear the role of PPV2 infection in PRDC as well its association with other infectious agents ([27] and references therein). Interestingly, in our study post-weaning pigs and piglets have lower PPV2 Ct values indicative of a higher PPV2 viral load compared to adults. These data differ from what was reported by Kim and coauthors, where the authors suggested that common vaccination protocols for sows and the passive immunity transferred to their progeny leads to a low viral load in piglets and a general increase in sows [66]. In our cases, adults have been vaccinated against PPV so we can assume a wave of viral spread and infection with higher prevalence and viral load in younger pigs and an efficient immune response against the virus in the oldest analyzed animals.

This is the first report of the presence of PPV2 in Sardinia and one of the few in Europe [67]. Further studies are necessary to clarify the significance of this novel emerging virus and its role in the clinical picture.

Briefly, virus coinfections are more recurrent in swine farms than single infections and PRDC falls under this condition. Thus, the mechanisms driving interactions between viruses are not refined in swine respiratory disease. A clear association between pathological findings and the presence of the virus infection would benefit the research community’s understanding of the interactions between viruses.

## 5. Conclusions

Swine respiratory diseases appear at a high prevalence in the pig population, posing an important threat to herd health. Nevertheless, it is critical to identify the pathogens implicated in PRDC for the application of effective control measures and prevention. This study suggests that PCV2 and PRRSV are associated with interstitial pneumonia and that TTSuV may also play a role in lung lesions. Further studies, using additional techniques such as immunohistochemistry and in situ hybridization, are necessary to understand the clinical significance and pathogenicity of emerging viruses such as PCV3 and PPV2, as well as to define the role of each virus in the development of PRDC and their actual impact on swine health.

## Figures and Tables

**Table 1:** Primers and probes used for the detection of swine viruses.

Virus	Primers and Probes	Sequence	Reference
PCV2	P1570F	5′-TGGCCCGCAGTATTCTGATT-3′	Opriessnig et al., 2003 [42]
	P1642R	5′-CAGCTGGGACAGCAGTTGAG-3′	
	P1591 probe	5′-CCAGCAATCAGACCCCGTTGGAATG-3′	
PCV3	PCV3 _353F	5′-TGACGGAGACGTCGGGAAAT-3′	Franzo et al., 2018 [43]
	PCV3_465R	5′-CGGTTTACCCAACCCCATCA-3′	
	PCV3_418probe	5′-GGGCGGGGTTTGCGTGATTT-3′	
TTSuV	QCOMF	5′-CGAATGGYWGAGTTTWYGCCGC-3′	Brassard et al., 2009 [44]
	QCOMR	5′-GCCCGAATTGCCCCTWGACTKCG-3′	
	QCOM probe	5′-CTCCGGCACCCGCCCAG-3′	
PPV2	PPV2DF	5′-TACTGAGCCCTAAGACTGACTACAAGC-3′	Xiao et al., 2013 [46]
	PPV2DR	5′-GTTTGTCTCGTTGTTCGTCTGATG-3′	
	PPV2D probe	5′-AACTGCTACATGAACCACTTTACCCCSTC-3′	

**Table 2:** Primers used for the sequencing of emerging viruses.

Virus	Primer Sequence	Reference
PPV2	PPV2-F6 GCTTTCTAGTCGGACCGGAAGT PPV2-R871 GCTCGGCCTTTCACGGTGGGC	Qin et al., 2018 [9]
TTSuV	TTSuV1_F CGGGTTCAGGAGGCTCAAT TTSuV1_R GCCATTCGGAACTGCACTTACT TTSuV2_F TCATGACAGGGTTCACCGGA TTSuV2_R CGTCTGCGCACTTACTTATATACTCTA	Blois et al., 2014 [47]
PRRSV	ORF7f 5′-GCCCCTGCCCAICACG-3′ ORF7r 5′-TCGCCCTAATTGAATAGGTGA-3′	Oleksiewicz et al., 1998 [48]

**Table 3:** Number of affected animals, mean (± standard deviation) and range of Ct values of viruses quantified in lungs of different swine categories.

Virus						
		PCV3	PPV2	TTSuV	PRRSV	PCV2
Categories						
A		1/10	10/10	10/10	2/10	10/10
Mean Ct (±SD) range		19.91	27.29 ± 1.12 24.7–28.64	25.38 ± 3.10 21.55–31.35	36.59 ± 0.41 36.3–36.89	27.22 ± 4.43 21.91–37.04
PW		2/10	5/0	10/10	2/10	3/10
Mean Ct (±SD) range		29.07 ± 12.15 20.48–37.66	21.21 ± 5.47 12.29–27.22	29.39 ± 2.98 25.89–35.33	34.30 ± 4.35 31.22–37.38	24.05 ± 9.7 17.49–35.19
P		4/9	8/9	9/9	3/9	2/9
Mean Ct (±SD) range		35.59 ± 4.88 28.47–39.18	23.06 ± 4.83 16.94–30.89	30.13 ± 5.32 24.58–40.18	37.38 ± 2.13 35.53–39.71	29.67 ± 8.84 23.41–35.92

Keys. A: adult pigs, PW: post-weaning pigs, P: piglets.

## Data Availability

Not applicable.
